# Centimeter-size achromatic metalens in long-wave infrared

**DOI:** 10.1515/nanoph-2024-0716

**Published:** 2025-03-13

**Authors:** Fen Zhao, Changchun Zhao, Yuqing Zhang, Jie Chen, Shaoqi Li, Wangzhe Zhou, Chongchong Ran, Yongcan Zeng, Huan Chen, Xin He, Jiagui Wu, Gangyi Zhu, Junbo Yang

**Affiliations:** College of Science, 58294 National University of Defense Technology, Changsha 410073, China; School of Artificial Intelligence, Chongqing University of Technology, Chongqing 401135, China; School of Physical Science and Technology, Southwest University, Chongqing 400715, China; College of Integrated Circuit Science and Engineering, Nanjing University of Posts and Telecommunications, Nanjing, 210003, China

**Keywords:** phase dispersion, centimeter-size, achromatic, thermal imaging

## Abstract

Metalens has shown its significantly ultra-light and ultra-thin features. However, large-aperture achromatic metalens is constrained by both maximum dispersion range and computational memory. Here, we propose a fully device optimizing framework that engineers phase dispersion and amplitude transmittance to create centimeter-size achromatic metalens operating in long-wave infrared regime (8–12 μm). Via wrapping group delay within a defined range and optimizing dispersion phase of desired wavelengths, chromatic aberrations can be effectively corrected. We verify our design by characterizing all-silicon 3.18-cm-diameter and 6.36-cm-diameter LWIR achromatic metalenses. Diffraction-limited tight-focusing can be achieved, and the normalized focal length shift is less than 3.3 × 10^−4^. Thermal imaging performance is verified on targets of holes or letters with a diameter or line width exceeding 2 mm. These findings facilitate the development of large-aperture achromatic metalenses and open up possibilities for lightweight imaging systems in long-wave infrared.

## 1 Introduction

**Figure 1:**
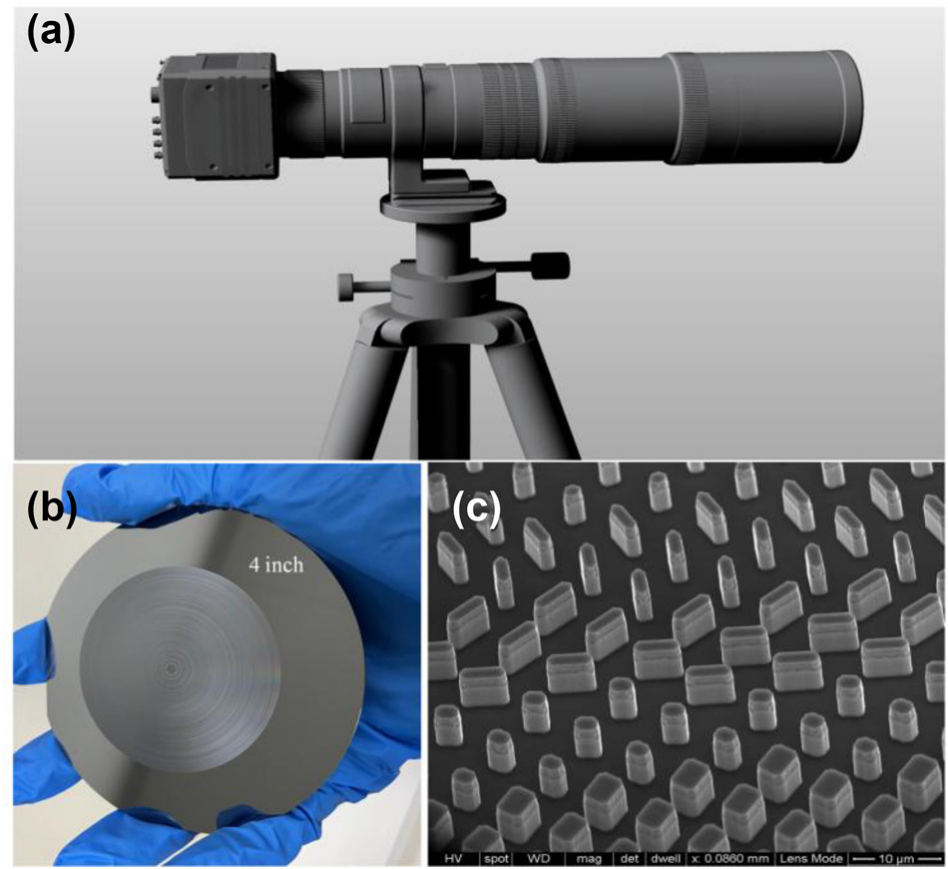
Cascade lens or metalens in Thermal Imaging Optics. (a) Refractive optics for high-resolution thermal imaging. (b) Optical image of large-aperture LWIR metalenses, and (c) sub-wavelength structures arranged on the surface.

Long-wave infrared (LWIR) 8–12 μm is one of the atmospheric transparency window, facilitating its penetration through the atmosphere for long-range detection and clear imaging, effectively addressing challenges presented by adverse weather conditions. Infrared thermal imaging is a critical key technology for noncontact thermography, target detection, identification, and tracking, due to its ability to accurately capture the thermal radiation characteristics of targets [1], [2], [3]. High-resolution imaging in LWIR typically requires bulky and precisely engineered refractive surfaces (Figure 1(a)), which ultimately add to the overall volume and weight of the optical system, especially for high numerical aperture (NA) or large-scale optics [4]. Additionally, refractive infrared lenses primarily rely on materials such as germanium, zinc selenide, and zinc sulfide, etc. Processing complexity and high costs associated these materials severely hinder the integration and large-scale production.

Metasurfaces offer a unique platform to precisely control optical wavefronts [5], [6], [7], [8], which have the potential to substantially reduce the size and complexity of imaging systems. One particular device that has attracted considerable attention is the planar lens (metalens) [9], [10], [11], which overcomes many limitations of traditional refractive optical components for ultra-light and ultra-thin platform. Owing to their unique optical modulation capabilities and advantages [12], metalenses have been found in a wide range of applications in optical imaging [13], [14], [15], sound capture [16], and electric field detection [17], [18]. Various metalenses operating in long-wave infrared have been demonstrated [19], [20], [21], unfortunately, they inevitably suffer from poor imaging quality due to their strong chromatic aberrations. Several solutions are currently available to address chromatic aberration in metalenses, including multi-wavelength achromatic metalenses [22], [23], multi-wavelength super-oscillation achromatic metalenses [24], [25], and broadband achromatic metalenses [26], [27], [28]. Nevertheless, these strategies encounter a shared hurdle [29], [30], [31], where achromatic metalenses have limited apertures due to maximum group delay achievable with meta-atoms. Achromatic metalenses with larger diameters and higher NA requires a larger group delay range supported by increasing the refractive index of the materials and the height of the meta-atoms, which usually result in extremely large aspect ratio and might not be available through present microfabrication technology. In extreme dispersion scenarios, low amplitude transmittance of meta-atoms can sharply reduce the focusing efficiency of achromatic metalenses, significantly hindering their practical application in large-aperture systems [32]. Although multizone dispersion engineering offers a potential avenue to surpass the constraints of the maximum dispersion range at discrete wavelengths [31], it is still a challenge to ensure uniform hot spots with consistently high focusing efficiency across all sampled wavelengths. Specifically, maintaining relatively stable peak intensities across varying wavelengths proves to be a persistent obstacle. In addition, metalenses have been limited by the scaling challenge of producing vast numbers of precisely engineered elements over a large area [33], [34], [35], [36], and full-wave simulation for large-aperture is also a computationally prohibitive problem [4]. Generally, achromatic metalenses have been limited to millimeter sizes. Although some researchers have enlarged the aperture of a single metalens to the centimeter sizes or even 10 cm, simultaneously eliminating chromatic aberration is unavailable (see Table S1 in Note S1, Supporting Information)

In this work, we present a general design principle to achieve large-aperture achromatic metalenses by wrapping group delay to multiple regions with a defined range and then optimizing phase discontinuity at the zone boundaries. And amplitude modulation is employed to boost the focusing efficiency of the lens and minimize variations in focal spots across different wavelengths. During the iterative optimization process, target optical field is computed by using vector angular spectrum method (VASM) with 2D Fourier transform accelerated operation. We demonstrate 3.18-cm-diameter and 6.36-cm-diameter LWIR achromatic metalenses, with a *F* number of 1 (NA = 0.45) in an all-silicon platform, and further evaluate their focusing and infrared thermal imaging capabilities.

## 2 Theoretical consideration

Large-aperture LWIR metalenses, composed of vast subwavelength structures, offer a practical solution for lightweight thermal imaging system (Figure 1(b) and (c)). Nevertheless, significant chromatic aberrations restrict their imaging performance. Dispersion manipulations have also been demonstrated in achromatic metalenses with diffraction-limited performance for visible, near-infrared, and THz regimes. These approaches do not break the limit of the maximum dispersion range of the structures. It is still a challenge to access large-aperture LWIR achromatic metalenses with high efficiency and low wavelength dependence. The key to achieve this goal is a fully device optimized design framework that modulates phase dispersion and amplitude transmittance to attain uniform focusing for all the desired wavelengths within the bandwidth at the same plane. However, designing a large-aperture (i.e. cm-size) metalens is a computationally problem, and full-wave simulation is not possible. We solve this problem by VASM and GPU-accelerated computing to simulate the light propagation, where Fourier transform is executed with CUDA functions from NVIDIA graphics card.

**Figure 2:**
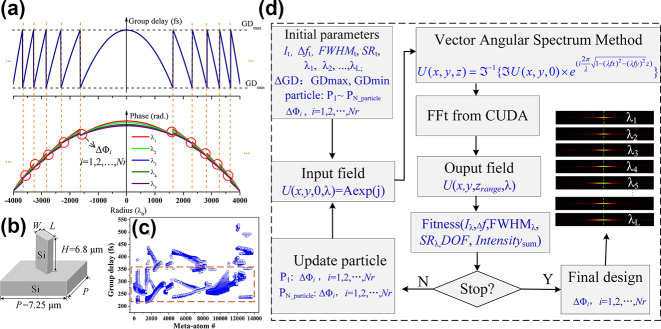
Design methodology to create large-aperture LWIR achromatic metalenses. (a) Schematic drawing of folded group delay and the corresponding phases for different wavelengths. Phase discontinuities at zone boundaries are marked by red circle. (b) Opted meta-atom of silicon pillar on a silicon substrate. The size of the meta-atom is *P* × *P*, the length is *L*, the width is *W*, and the height is *H*, respectively. (c) Group delay distribution of the meta-atoms from the created library, where meta-atoms with polynomial fitting coefficient greater than 0.99 within the concerned bandwidth are marked by the orange dashed box. (d) Block diagram of the optimization routine leveraging GPU-accelerated computing.

Considering typical phase modulation of wrapping the phase profile to 2π range, we attempt to directly fold the required group delay of the metalenses within a defined range. To match the maximum group delay (GD) range of the structures, we fold the required group delay of an achromatic metalens into a defined range, generating *Nr* zones along the radius as illustrated in Figure 2(a) (top). In detail, GD_min_ and GD_max_ are the minimum and maximum group delays, which are bounded by the meta-atom library. Since group delay discontinuity at the boundary between (*i* − 1)th and *i*th zone destroys constructive interfere in dispersion engineering, for the designed wavelength of *λ*_0_ = 10.6 µm (i.e., angular frequency of *ω*_0_ = 177.82 rad/ps), we introduce additional phase ΔΦ_*i*_ at the zone boundaries. Phase profile of the dispersion-engineered metalens in the *i*-th zone is as follows [32]

(1) $$\begin{array}{ll} \Phi_{i}\left(\omega_{0},r\right) & =2\pi n-\frac{\omega_{0}}{c}\\ & \;\times \left(\sqrt{f{\left(\omega_{0}\right)}^{2}+r^{2}}-\sqrt{f{\left(\omega_{0}\right)}^{2}+{R_{\text{lens}}}^{2}}\right)\\ & \;+\Delta \Phi_{i} \end{array}$$

where *r* is the radial coordinate, *f*(*ω*_0_) is the focal length at the designed angular frequency, *R*_lens_ is the lens radius, and *n* is the integer. The phase distribution within *i*-th (*i* = 1, 2, …, *Nr*) zone at a certain frequency *ω* is related to the center frequency *ω*_0_ according to Eq. (2) [27] as described in Figure 2(a) (bottom).

(2) $$\begin{array}{ll} \Phi_{i}\left(\omega ,r\right) & =\Phi_{i}\left(\omega_{0},r\right)+\frac{\partial \Phi_{i}\left(\omega ,r\right)}{\partial \omega }\left(\omega -\omega_{0}\right)\\ & \;+\frac{\partial^{2}\Phi_{i}\left(\omega ,r\right)}{2\partial \omega^{2}}{\left(\omega -\omega_{0}\right)}^{2} \end{array}$$

This phase profile can be realized by using spatially varied meta-atoms that can independently control phase and dispersion (group delay $\frac{\partial \Phi_{i}\left(\omega ,r\right)}{\partial \omega }$ and group delay dispersion $\frac{\partial^{2}\Phi_{i}\left(\omega ,r\right)}{2\partial \omega^{2}}$ ). Group delay is majorly concerned in achromatic design due to the negligible quadratic term. Here, an all-silicon platform composed of silicon pillars on a silicon substrate is opted shown in Figure 2(b), which is beneficial to simplify the fabrication process. The refractive index of Si pillars is almost kept at a constant value of 3.42 as the wavelength changes from 8 to 12 µm [37]. A library of 1.4 × 10^4^ different meta-atoms are created within the operation bandwidth by varying their geometric parameters, including the length, width, height, and the pitch of the meta-atoms within the fabrication limited bounds. For a given height of *H* = 6.8 µm, the optimized pitch, *P* is 7.25 µm. The GD of the retained meta-atoms ranges from 212 to 532 fs (Figure 2(c)), whose polynomial fitting coefficient are greater than 0.99 within the concerned bandwidth.

The introduced additional phases ΔΦ_*i*_ destroy the perfect focusing of hyperbolic phase metalens, and Particle Swarm Optimization (PSO) is utilized to optimize the phase discontinuities at each zone boundary. We define the fitness function FOM by evaluating the difference between the actual optical field and the preset optical field in the optimization process.

(3) $$\begin{array}{ll} FOM & ={\left[\delta_{1}\cdot \frac{abs\left(I_{\min}-I_{t}\right)}{I_{t}}\right]}^{2}+{\left[\delta_{2}\cdot \frac{\left(\Delta f_{\max}-\Delta f_{t}\right)}{\Delta f_{t}}\right]}^{2}\\ & \;+{\left[\delta_{3}\cdot \frac{\left(FWHM_{\max}-FWHM_{t}\right)}{FWHM_{t}}\right]}^{2}\\ & \;+{\left[\delta_{4}\cdot \frac{\left(SR_{\max}-SR_{t}\right)}{SR_{t}}\right]}^{2}+{\left[\delta_{5}\cdot stdev\left(DOF\right)\right]}^{2}\\ & \;+{\left[\delta_{6}/\min\left(Intensity_{\mathrm{sum}}\right)\right]}^{2} \end{array}$$

Δ*f*_max_, *FWHM*_max_, and *SR*_max_ are the minimum peak intensity of the focal spots, the maximum normalized focal length shift (i.e., the actual focal length deviating from the preset value), the maximum full-width-at-half-maximum (*FWHM*) and the sidelobe ratio for all the wavelengths within the bandwidth, respectively, while *I*_*t*_, Δ*f*_*t*_, *FWHM*_*t*_, and *SR*_*t*_ are the target parameters. The standard deviation of depth of focus (DOF) for different wavelengths and intensity sum (*Intensity*_sum_) of 10 sampling points near the focal plane along *z*-axis are used as an evaluation metric in tight-focusing. *δ*_1_, *δ*_2_, *δ*_3_, *δ*_4_, *δ*_5_, and *δ*_6_ are the weights of concerned parameter, respectively. The optimization goals are maximizing the light intensity at the design focal spots and suppressing spurious focusing elsewhere along the optic axis to achieve high efficiency. The optimization routine halts when the value of FOM converges or ceases to decrease, thus constraining our metalenses to realize achromatic focusing for the sampled wavelengths (i.e., *λ*_1_, *λ*_2_, …, *λ*_*L*_) within the bandwidth (Figure 2(d)).

## 3 Large-aperture LWIR achromatic metalenses

Using the above framework, we designed LWIR achromatic metalenses with a diameter of 3.18 cm and 6.36 cm (i.e., 3000*λ*_0_ and 6000*λ*_0_), and a *F* number of 1. For left-circularly polarized (LCP) incident waves impinge on the meta-atoms from the substrate side, the phase shift of the transmitted right-circularly polarized (RCP) waves can be continuously tuned by rotating the Si blocks around the *z*-axis. Each meta-atom partially converts LCP light to RCP light, and vice versa. This polarization conversion from LCP to RCP and RCP to LCP can be described by the Jones’ matrix as follows [38]

(4) $$\begin{array}{ll} \left[\begin{array}{c} E_{\text{LCP}}^{\text{out}}\\ E_{\text{RCP}}^{\text{out}} \end{array}\right] & =\left[\begin{array}{cc} \left(t_{L}+t_{s}\right)/2 & \left(t_{L}-t_{s}\right)e^{-2i\theta }/2\\ \left(t_{L}-t_{s}\right)e^{+2i\theta }/2 & \left(t_{L}+t_{s}\right)/2 \end{array}\right]\\ & \;\times \left[\begin{array}{c} E_{\text{LCP}}^{in}\\ E_{\text{RCP}}^{in} \end{array}\right] \end{array}$$

where *t*_*L*_ and *t*_*s*_ are complex transmission along long and short axis, respectively, *θ* is the rotation angle of meta-atoms, “out” means output field, and “in” means input field. The phase profiles of the lens for different wavelengths are realized by utilizing various Si-blocks with suitable rotate-angle, roughly *ϕ* = 2*θ*, where the relative phase delay between meta-atoms has been considered.

This design framework is well suited for obtaining broadband performance; however, the calculation is limited to several sampled wavelengths spanning the bandwidth due to memory constraints. Three 3.18-cm-diameter achromatic metalenses have been designed for 3-, 5-, and 9-sampled wavelengths within 8–12 μm, and the relative group delay in each zone is bounded by 140 fs (femtosecond) as marked by red dashed box in Figure 2(c). The achromatic performance of the proposed metalens has been investigated by using VASM with 2D Fourier transform accelerated operation (see Figure S1 in Note S2, Supporting Information). It can be seen that the actual focal point at the position of the maximum intensity is almost the same for all simulated wavelengths, which shows an excellent achromatic focusing performance. Some extra focal spots deviating from the preset focal plane can be observed along the optical axes with lower iterations (see Figure S1(a) in Note S2, Supporting Information), and tight-focusing hot spots will be achieved for five sampled wavelengths after optimizing the weights and increasing the iterations (see Figure S1(b) in Note S2, Supporting Information). Furthermore, simulated results of the achromatic metalens for nine sampled wavelengths are also presented (see Figure S1(c) in Note S2, Supporting Information), and almost all the waves with different wavelengths constructively interfere at the observation plane. Our metalenses have a uniform performance for the sampled wavelengths with explicitly defining uniformity (i.e., DOF and *Intensity*_*sum*_) as optimization criteria. It is noted that more sampled wavelengths within the bandwidth make the peak intensity of the focal spots much lower, and more optimization time will help to improve the relative intensity of the metalens. Specifically, the average transmittance of silicon wafer in LWIR is relatively low [38], [39], we set its initial value in each ring-belt of 0.66 in the optimization, and it will be replaced with the real value of the selected meta-atom.

**Figure 3:**
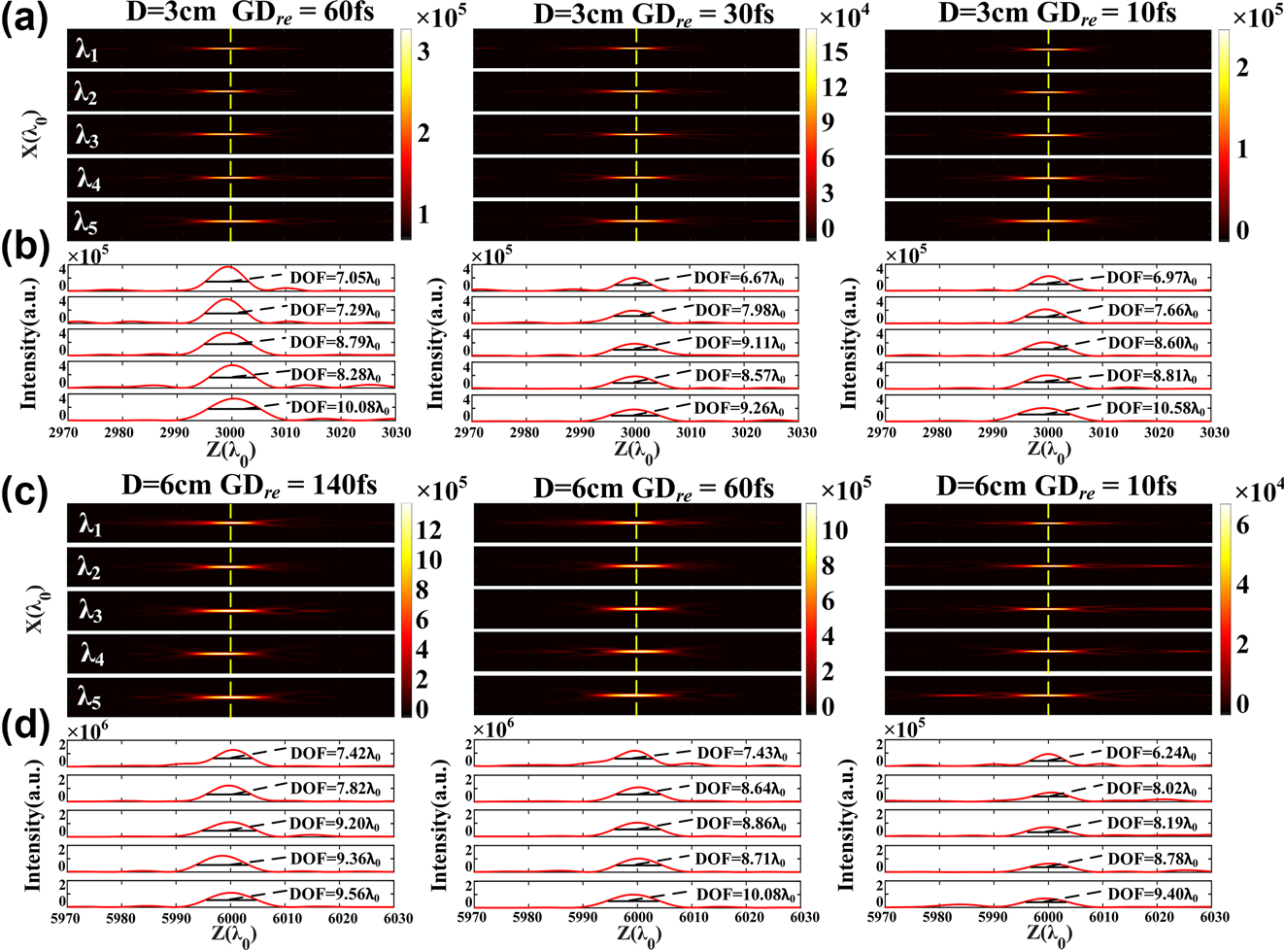
Simulated results of large-aperture achromatic metalenses. (a) 2D optical intensity profiles along the *z*-axis of three 3.18-cm-diameter metalenses with relative group delay bounded by 60, 30, and 10 fs, respectively. (b) Intensity distribution curves in the *z*-axis crossing the center of each focal spot. The depth of focus for different wavelengths is also marked. (c–d) 2D optical intensity profiles and intensity distribution curves of 6.36-cm-diameter achromatic metalenses with relative group delay bounded by 140, 60, and 10 fs, respectively. The five sampled wavelengths are 8 μm, 9 μm, 10.6 μm, 11 μm, and 12 μm, respectively.

To better understand the dispersion-engineered effect, 3.18-cm-diameter and 6.36-cm-diameter achromatic metalenses with different group delay distributions are demonstrated. Figure 3(a) gives the 2D optical intensity profiles along the *z*-axis of three 3.18-cm-diameter metalenses, and the relative group delay (GD_*re*_ = GD_max_ − GD_min_) in each zone is bounded by 60, 30, and 10 fs, respectively. The actual focal length almost keeps at the preset value of 3000*λ*_0_ as marked by the yellow-dashed lines, and the normalized focal length shift Δ*f* = (*f*_actual_ − *f*_preset_)/*f*_preset_ is less than 3.3 × 10^−4^. The *FWHM* values of all the spots located at the focal plane are closed to the diffraction limit of 1.118*λ*_0_ (i.e., 0.5*λ*_0_/NA). Our metalenses show uniform focusing performance for the sampled wavelengths, and the peak intensity decreases for the relatively small GD_*re*_ unless costing more time. The intensity distribution curves in the *z*-axis crossing the center of each focal spot are presented in Figure 3(b), and the peak intensity are observed near the focal plane, which gives clear evidence for achromatic focusing of the designed metalenses. The depth of focus for different wavelengths is also marked beside the curves, which are slightly larger than the ideal result of 5*λ*_0_ (i.e., *λ*_0_/NA^2^). Figure 3(c) and (d) depicts the 2D optical intensity profiles and intensity distribution curves of 6.36-cm-diameter achromatic metalenses, and the relative group delays are bounded by 140, 60, and 10 fs, respectively. Similar achromatic performance is achieved comparing with the 3.18-cm-diameter achromatic metalenses, indicating that our method has a potential to realize any large-aperture achromatic metalens.

**Figure 4:**
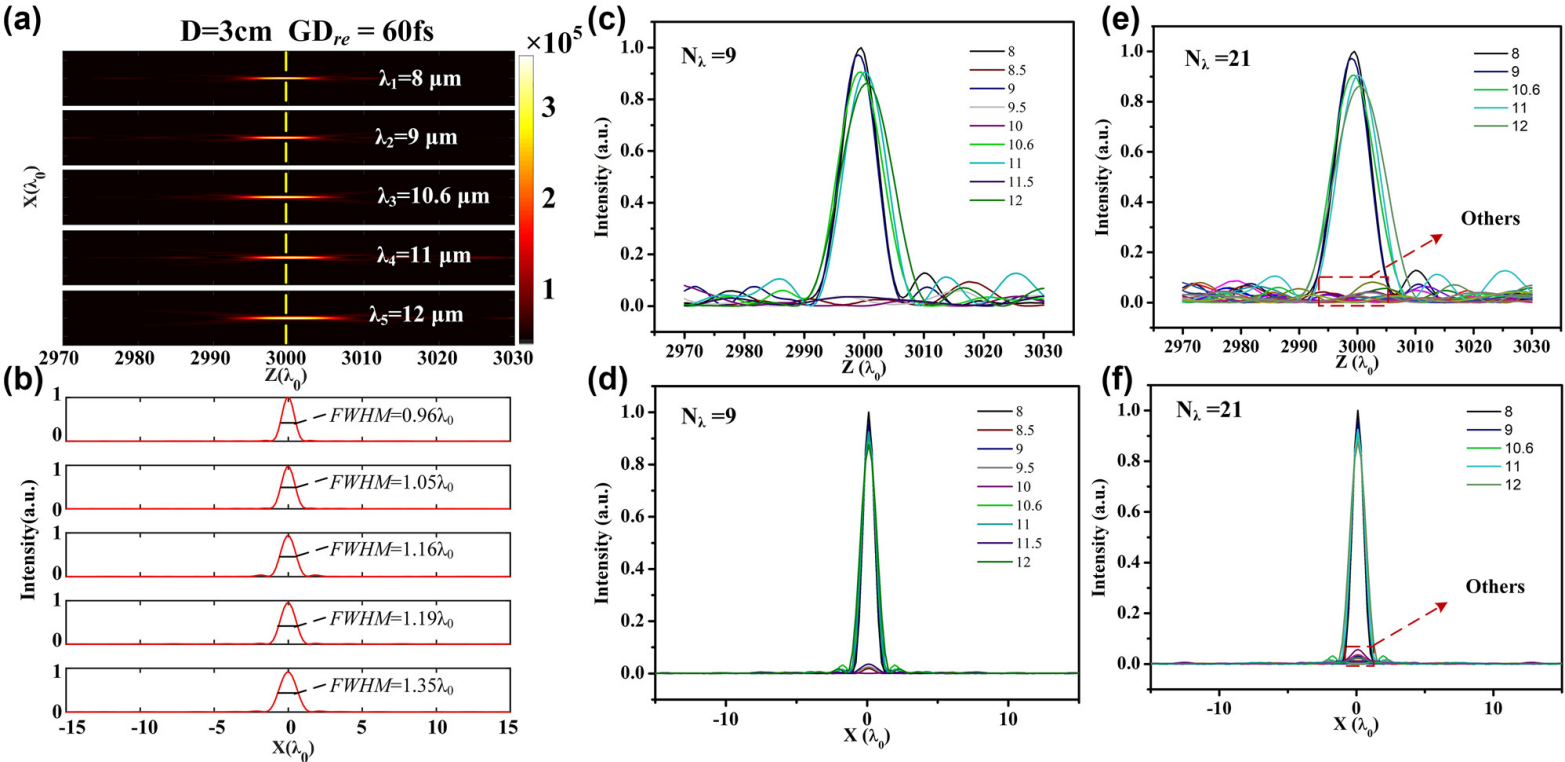
Achromatic and filtering performance of the designed metalens. (a) 2D optical intensity profiles at the propagation plane of the 3.18-cm-diameter metalenses with relative group delay of 60 fs. (b) Intensity distribution curves in the *x*-direction crossing the center of each spot located at focal plane as marked by yellow dashed line in (a). Corresponding intensity distribution curves (c) along the optic axis and (d) at the preset focal plane for the nine wavelengths. (e–f) Corresponding intensity distributions for the 21 different wavelengths with an interval of 0.2 μm within 8–12 μm.

We further verify the achromatic performance of the designed metalenses. For a 3.18-cm-diameter metalenses with relative group delay of 60 fs, its intensity profiles along propagation direction for the five sampled wavelengths are exhibited in Figure 4(a). Constructive interference is realized in the axial calculated region, and no more obvious subspots can be observed. Figure 4(b) gives the corresponding intensity distribution curves in the *x*-direction crossing the spots’ center, and the values of *FWHM* varies from 0.96*λ*_0_ to 1.35*λ*_0_, which are closed to the diffraction limit. Phase profiles of the metalens for nine different wavelengths within the bandwidth are obtained according to Eq. (2), and its intensity distribution curves along the optic axis are depicted in Figure 4(c). Obviously, only the five sampled wavelengths contribute to the optical field, showing a natural filtering effect. The corresponding intensity distributions of the metalens at the preset focal plane are presented in Figure 4(d), and incident light deviating from the sampled wavelength has a negligible effect to the focusing performance, where the peak intensities of the spots for other wavelengths decrease rapidly to less than 0.25 %. Similar results can be obtained when the number of simulated wavelengths is extended to 21 with an interval of 0.2 μm within 8–12 μm as illustrated in Figure 4(e) and (f), where the peak intensities decrease rapidly to less than 0.29 % for other wavelengths. Our approach enables the realization of achromatic metalenses for nonuniformly sampled wavelengths within a bandwidth. Impact of beams deviating from the sampled wavelengths on the optical field will be naturally suppressed or filtered out, without an extra a band-pass filter [40]. This is favorable for customized achromatic design, which is crucial for high-quality imaging.

**Figure 5:**
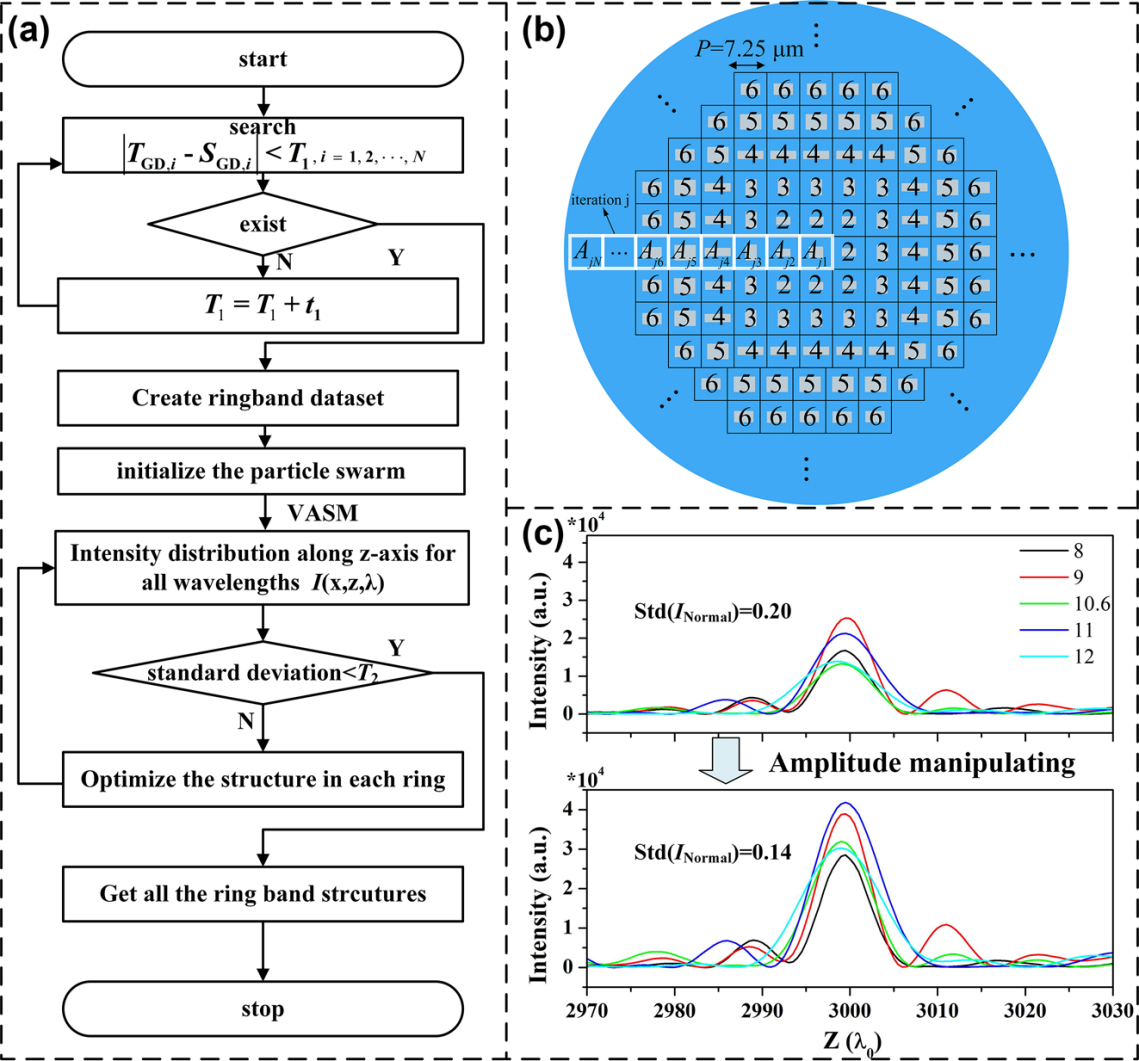
Optimization design method for amplitude modulation of achromatic metalenses. (a) Flow chart of dispersion and amplitude engineering by matching the meta-atoms in each belt. (b) Iterative refinement of the arrangement on lens’ surface. (c) Intensity curves of the metalens for the sampled wavelengths pre- and post-amplitude modulation.

In fact, the amplitude transmittance of dispersion-engineered meta-atoms varies noticeably, leading to a notable wavelength-dependent focusing efficiency of the metalens, implying that different wavelengths correspond to significant variations in focusing efficiency. Our previous research indicates that modulating the amplitude transmittance of each ring-belt in the metalens surface can effectively tune the focusing spot at a specific wavelength [32]. To keep the focal length shift and depth of focus of the metalens unchanged, maximize its focusing efficiency, and minimize wavelength dependence as much as possible, the amplitude transmittance of each ring-belt on the surface is further adjusted utilizing particle swarm optimization (PSO) algorithm, as illustrated in Figure 5(a). For dispersion engineering, a suitable matching error is set to select meta-atoms arranging on the lens surface, which represents the variance between the theoretical group delay and the actual value of the matched meta-atom. This process generates unit cell data at each ring-belt of the metalens, namely ring-belt database. It is noted that certain ring-belts may accommodate multiple meta-atoms that fulfill the dispersion criteria. *T*_*GD*,*i*_ represents the theoretical group delay (GD) for the *i*-th ring belt, while *S*_*GD*,*i*_ denotes the simulated GD value corresponding to the meta-atoms. *T*_1_ indicates the matching error of GD. For the case of the *i*-th ring-belt (*i* = 1, 2, …, *N*), if no suitable meta-atoms can be found in the library, an additional error *t*_1_ is added to the matching error. This process persists until all ring-belts are equipped with appropriately matched meta-atoms. Meta-atoms are randomly selected from the ring-belt database and placed on the lens surface. If the standard deviation of the relative spot intensity, as computed by VASM, exceeds *T*_2_, the structures on each ring-belt undergo iterative refinement (Figure 5(b)). The optimization goal is to ensure that the metalens maintains high focusing efficiency while achieving uniform intensity of focusing spots for all sampled wavelengths.

For the 3.18-cm-diameter achromatic metalenses with the relative group delay of 140 fs, the above amplitude modulation can effectively improve the focusing efficiency and minimize the wavelength dependency of the focusing spots, as depicted in Figure 5(c). Specifically, the metalens is composed of the first matched meta-atoms selected from the ring-belt database with the initial matching error of *T*_1_ = 0.5. The peak intensities for the five designed wavelengths (i.e., 8 μm, 9 μm, 10.6 μm, 11 μm, and 12 μm) exhibit notable variations as shown in the top layer of Figure 5(c), with the maximum relative intensity at approximately 2.5 × 10^4^, a normalized standard deviation of 0.20. The simulated intensity distributions on the focal plane were used to calculate the focusing efficiency, which are 11.8 %, 15.9 %, 9.2 %, 14.2 %, and 10.5 %, respectively. After amplitude modulation, the disparities in peak intensities for the five wavelengths diminish as depicted in the bottom layer, with the maximum relative optical intensity hovering around 4 × 10^4^, normalized standard deviation of 0.14, and focusing efficiency of 14.2 %, 17.6 %, 11.9 %, 18.1 %, and 14.2 %, respectively. Clearly, the amplitude modulation significantly boosts the focusing efficiency of the metalens and mitigates the wavelength dependency of the focal spots. In this work, 3.18-cm-diameter and 6.36-cm-diameter high efficiency achromatic metalenses with amplitude-modulation will be fabricated for characterizing their chromatic aberration performance, where the relative group delay is a constant (GD_*re*_ = 140 fs). For the 3.18-cm-diameter metalens, the entire surface is divided into 2,194 concentric rings, 88 zones are created according to the optimization results, and 47 kinds of meta-atoms are selected for arranging within these zones. The major parameters involving the length and width are listed in Table S2 (see Table S2 in Note S2, Supporting Information). In addition, metalens with other size can be also achieved by selecting meta-atoms from the library.

Essentially, our design method is not limited by the polarization state of incident light. A LWIR polarization-insensitive achromatic metalens is demonstrated with a diameter of 6000*λ*_0_ (i.e., 3.18 cm) and *F* number of 1. A library of Si meta-atoms is created by varying their geometric parameters, i.e., length *L* of square, radius *R* of circle, and *R*_in_ and *R*_out_ of ring. These polarization-insensitive structures finely arranged on the metalens’ surface, and its optical intensity profiles exhibit clear achromatic focusing for the 6-sampled wavelengths (i.e., 8, 9, 10, 10.6, 11, 12 μm), maintaining a normalized focal length shift less than 2.5 × 10^−4^ (see Figure S2 in Note S2, Supporting Information). The major parameters of partial meta-atoms are listed in Table S3, and the phase and amplitude transmittance distributions are given in Figure S3 (see Table S3 and Figure S3 in Note S2, Supporting Information).

To experimentally demonstrate the proposed LWIR achromatic metalenses, four 3.18-cm-diameter and a 6.36-cm-diameter samples on a single wafer are fabricated by inductively coupled plasma (ICP) etching in an all-silicon platform (see Figure S4 in Note S3, Supporting Information), where patterning is accomplished with the help of SiO_2_ mask. Optical images of the entire metalenses with diameters of 3.18 cm and the zoom-in images marked by the red dashed box are depicted in Supporting Information (see Figure S5 in Note S3). For the 6.36-cm-diameter metalens, we have captured magnified views near its center and edge using a microscope with a 50-fold magnification (see Figure S6 in Note S3, Supporting Information). Notably, the meta-atoms with diminutive dimensions tend to exhibit smoother profiles deviating from the design value, which will lead to adverse effects on the lens’ focusing and imaging capabilities.

**Figure 6:**
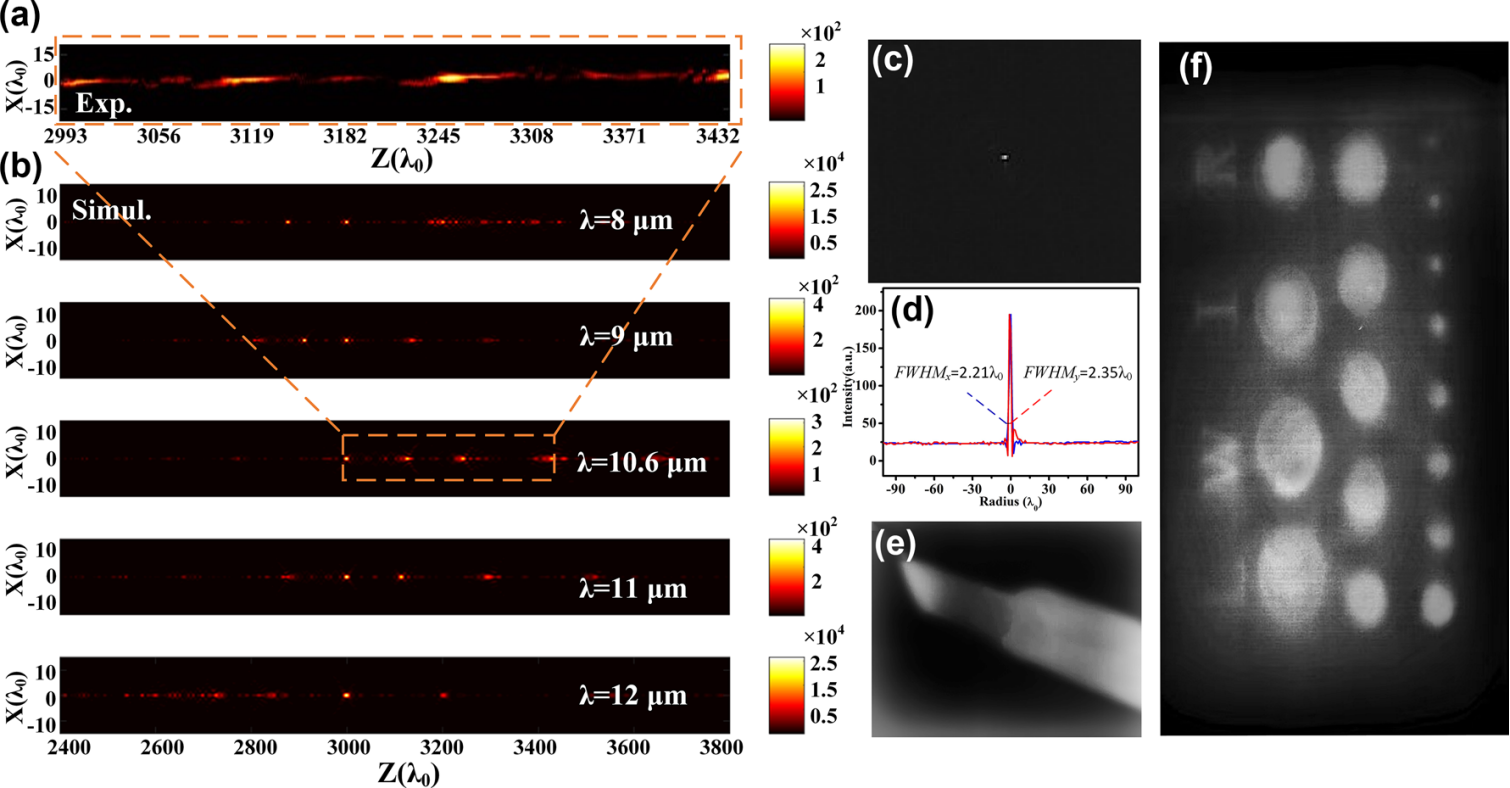
Characterization and Imaging Performance of the proposed metalens. (a) Experimentally measured intensity profile of the diffraction pattern on the propagation plane utilizing CO_2_ laser. (b) Simulated results of the metalens for the five sampled wavelengths, covering the experimentally measured range in the propagation plane. (c) 2D intensity distribution at the focal plane. (d) Intensity distribution curves in the *x*-direction (blue) and the *y*-direction (red) crossing the center of the focal spot. Imaging result of (e) electric soldering iron and (f) stainless sheet with holes and letters (“L,” “W,” “I,” “R”) with width of 2 mm.

To characterize the actual performance of the fabricated achromatic metalenses, a CO_2_ laser is used as light source with a center wavelength of 10.6 μm. The experimental setup and method can be found in Supporting Information (see Figure S7 in Note S3). As the linearly polarized wave is a superposition of the RCP wave and the LCP wave [41], [42], its LCP component can be focused by the proposed metalens. For a 3.18-cm-diameter achromatic metalens, its experimentally measured intensity profile of the diffraction pattern on the propagation plane behind the metalens is given in Figure 6(a). There are multiple discrete focal spots observed besides the preset spot within the range of 3.17–3.64 cm along *z*-axis, which is coincident with the simulated results as marked by orange dashed box in Figure 6(b). The first three focal spots are approximately uniformly spaced, and the fourth focal spot is relatively separated. Here, only the area of interest is shown, ensuring a high precision step of 2 μm in the experiment. It indicates that the capability to independently engineer phase profile, dispersion within each zone, and additional phase at zone boundaries can realize constructive interference in the targeted region along *z*-axis (i.e., *z*_*rang*_ = 60*λ*_0_) for the sampled wavelengths, as shown in Figure 3. In contrast, destructive interference occurs outside this region, and it can be mitigated by extending the *z*_*rang*_ during optimization. In fact, the presence of multiple focal points along the propagation plane holds significant value in various fields, such as optical coherence tomography, microscopic spectral tomography, three-dimensional reconstruction, etc. Obviously, the hot spot at the focal plane is much smaller than those in the other positions. Figure 6(c) gives the 2D intensity distribution at the focal plane, and the corresponding intensity distribution curves in the *x*-direction (blue) and the *y*-direction (red) crossing the center of the focal spot are presented in Figure 6(d). The sizes of the focal spot, i.e., *FWHM*_*x*_ and *FWHM*_*y*_, are 2.21*λ*_0_ and 2.35*λ*_0_, respectively, which are close to the Abbe diffraction limit of 1.12*λ*_0_.

Thermal imaging tests were performed on an electric soldering iron as depicted in Figure 6(e). Our metalens and the long wave infrared CCD were fixed on the upper and lower layer of a home-made mold, respectively, with a distance of 3.18 cm (equal to focal length of 3000*λ*_0_) between the two layers. And the target was placed in front of the metalens about 20 cm. Due to the relatively low efficiency of diffractive elements, objects with low spectral radiance emittance or temperature cannot be directly imaged. Therefore, we directly heated the electric soldering iron to 200 °C, whose spectral radiance emittance was much higher than that in room temperature within wavelength range of 8–12 µm according to the Planck function [39], [43].

(5) $$L_{b\lambda }\left(T\right)=\frac{c_{1}\lambda^{-5}}{\exp\left(\frac{c_{2}}{\lambda T}\right)-1}$$

where *T* is the temperature of the object, *c*_1_ = *2hc* ^2^ is the first radiation constant, *c*_2_ = *hc*/*k* is the second radiation constant, *c* is the speed of light in a vacuum, *h* is Planck’s constant, and *k* is Boltzmann’s constant. Thus, spectral radiance emittance of objects at different temperatures can be attained. A clear image can be captured by the infrared CCD, and the higher the temperature of the object, the sharper its contour under the same condition. This can be explained by the formula of spectral radiant power [44], where a higher object temperature leads to increased spectral radiant power and stronger signals injecting to detector, resulting in improved imaging quality. In addition, moving the iron horizontally parallel to the lens can also obtain clear images from −15 mm to 15 mm constrained by the range of translation stage. To further assess its imaging ability, a stainless sheet containing 17 circular holes of varying diameters (1 mm–17 mm) and four letters (“L,” “W,” “I,” “R”) with width of 2 mm was utilized as the target for image acquisition. A heating stage at 200 °C was used as the black body radiation source, and the stainless sheet was positioned directly on the stage with four small nuts separated at the four corners. Imaging results reveal that almost all the holes and letters can be resolved despite exhibiting low contrast for holes with diameter less than 2 mm and letters in the edge of the sheet as shown in Figure 6(f). In addition, the fabricated 6.36-cm-diameter achromatic metalenses are also measured by utilizing the same optical system. Due to the complexity of our achromatic metalenses, which consist of hundreds of different meta-atoms, it is challenging to maintain small geometric errors uniformly across the entire lens surface, constraining by present microfabrication technology. With the rapid advancement of optimal design methodologies and fabrication techniques, the realization of larger achromatic metalenses for high-quality infrared imaging without heating will be possible in the near future.

## 4 Conclusions

In this work, we have demonstrated universal fully device optimized framework for centimeter-size LWIR achromatic metalens with GPU-accelerated computing. According to the phase dispersion distribution of meta-atoms, achromatic performance can be obtained by wrapping the group delay of the metalenses within a defined range and optimizing additional phases only at the folding positions. By simultaneously controlling the phase, dispersion, additional phase, and amplitude in each ring-belt, constructive interference can be achieved within a defined axial range for all the desired wavelengths. This method is well suited for incident light of any polarization state. Moreover, chromatic aberrations of the metalens within the working bandwidth can be effectively corrected by filtering out the influence of beams deviating from the sampled wavelength. We experimentally demonstrate the tight-focusing and imaging capability of fabricated metalenses, and a heating electric soldering iron and stainless sheet can be clearly imaged.

This method breaks the limitations of maximum dispersion range, which holds significant value for realizing large-aperture achromatic metalenses. Expanding the axial range to encompass a broad region, multiple focal points emerge alongside the designated focal spot. By finely adjusting the relative phases of each zone at the propagation plane, axial focusing positions can be precisely controlled, which is crucial for microscopic spectral tomography [45], polarization detection [46], trapping and manipulation of nanoparticles [47], etc. Furthermore, the relative group delay can be set as needed, which is vital for enhancing the efficiency of the achromatic metalens, because more meta-atoms with high transmittance can be arranged on the lens’ surface for a smaller relative group delay. With the rapid progress of machine learning-enabled meta-optics design and innovative phase-engineered [48] as well as material layers engineered [49] for matching dispersion, the efficiency of centimeter-size LWIR achromatic metalens can be further improved, facilitating passive imaging in the real word.

## Supplementary Material

Supplementary Material Details
