# Supplementary material for: Centimeter-size achromatic metalens in long-wave infrared
Source: Nanophotonics. 2025 Mar 13;14(5):589–99. doi: 10.1515/nanoph-2024-0716 (PMC11953723; doi:10.1515/nanoph-2024-0716)
Supplement: Supplementary file 1 — Supplementary Material Details [file j_nanoph-2024-0716_suppl_001.docx]

Supporting Information

Centimeter-size Achromatic Metalens in Long-Wave Infrared

**Fen Zhao^1,4^, Changchun Zhao^4^, Yuqing Zhang^2^, Jie Chen^4^, Shaoqi Li^1^, Wangzhe Zhou^1^, Chongchong Ran^2^, Yongcan Zeng^2^, Huan Chen^1^, Xin He^1^, Jiagui Wu^2*^,Gangyi Zhu^3*^, Junbo Yang^1^***

*^1^College of Science, National University of Defense Technology, Changsha 410073, China*

^2^ School of Physical Science and Technology, Southwest University, Chongqing 400715, China

^3^College of Integrated Circuit Science and Engineering, Nanjing University of Posts and Telecommunications, Nanjing, 210003, China

^4^School of Artificial Intelligence, Chongqing University of Technology, Chongqing 401135, China

E-mail: [mgh@swu.edu.cn](mailto:mgh@swu.edu.cn)

E-mail:zhugangyi@njupt.edu.cn

E-mail: [yangjunbo@nudt.edu.cn](mailto:yangjunbo@nudt.edu.cn)

**Contents**

**Supplementary Note S1:** **Representative metalenses**

**Supplementary Note S2: Numerical simulations for the metalenses**

**Supplementary Note S3: Sample fabrication and measurement setup**

**Supplementary Note S1:** **Representative metalenses**

Table S1 lists some representative metalenses, including their diameter, operating bandwidth, material, numerical aperture, processing method, focusing efficiency, and other parameters. λ_0_ is the central working wavelength with unit in micrometers.

Table S1. Majors parameters of metalenses operating across the spectrum from visible to LWIR.

| Date | Diameter | λ_0_(μm) | Bandwidth | Material | NA | Fabrication | Focusing Efficiency |
| --- | --- | --- | --- | --- | --- | --- | --- |
| 2018 | 20 µm | 0.53 | 470-670 nm | TiO_2_ | 0.2 | EBL | ~20% ^[1]^ |
| 2021 | 2 mm | 0.532 | RGB | TiO_2_ | 0.3 | EBL | 11%,12%,10%^[2]^ |
| 2022 | 1 cm | 0.532 | RGB | TiO_2_ | 0.3 | RIE | 13%,15%,14%^[3]^ |
| 2020 | 2 mm | 0.94 | 0.94 | a-Si | 0.5 | ArF DUV | 29.2%^[4]^ |
| 2021 | 0.9 mm | 1.55 | 1.55 | Si | 0.04 | NIL | 26%^[5]^ |
| 2021 | 1.5 mm | 5.2 | 5.2 | GSST | 0.47 | EBL | 21.6%^[6]^ |
| 2024 | 5 cm | 10.6 | 10.6 | Si | 0.59 | stepper | *Not reported*^[7]^ |
| 2024 | 10 cm | 9.35 | 9.35 | Si | 0.89 | LDW | 41.22%^[8]^ |
| Ours | 3.18 or 6.36 cm | 10.6 | 8-12 µm | Si | 0.45 | ICP | ~15% |

**Supplementary Note S2: Numerical simulations for the metalenses**

Three 3.18-cm-diameter achromatic metalenses have been designed for 3-, 5- and 9-sampled wavelengths within 8-12 μm, and the relative group delay in each zone is bounded by 140 fs. Figure S1 gives the optical intensity profiles in the X-Z propagation plane at the sampled-wavelengths.


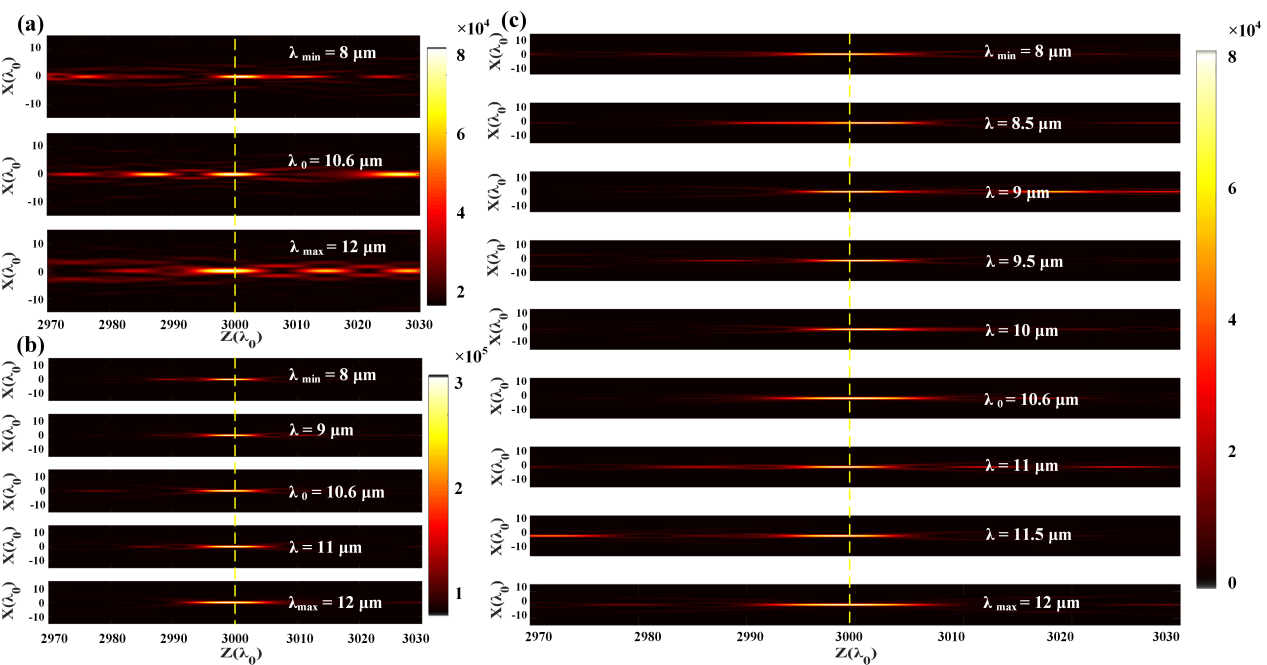


Figure S1. Simulated 2D optical intensity profiles of achromatic metalenses for (a) 3-, (b) 5- and (c) 9-sanmpled wavelengths within 8-12 μm. All sampled wavelengths are marked in the figure, and the yellow-dashed lines denote the preset focal plane.

Here, 3.18-cm-diameter and 6.36-cm-diameter high efficiency achromatic metalenses with relative group delay of 140 fs will be fabricated. As an example, Table S2 list the major parameters of the selected meta-atoms arranging on the 3.18-cm-diameter metalens’ surface. 6.36-cm-diameter metalens can be also achieved by selecting meta-atoms from the library.

| No. | *L*(μm) | *W*(μm) | GD(fs) | No. | *L*(μm) | *W*(μm) | GD(fs) |
| --- | --- | --- | --- | --- | --- | --- | --- |
| 1 | 3.84 | 4.82 | 132 | 25 | 1.9 | 4.19 | 45 |
| 2 | 2.77 | 4.3 | 93 | 26 | 1.9 | 5.47 | 44 |
| 3 | 3.02 | 4.99 | 92 | 27 | 1.9 | 5.55 | 43 |
| 4 | 3 | 4.97 | 91 | 28 | 2 | 5.33 | 41 |
| 5 | 2.73 | 4.24 | 81 | 29 | 2 | 5.41 | 40 |
| 6 | 2.71 | 4.2 | 74 | 30 | 2 | 5.49 | 38 |
| 7 | 2.69 | 4.2 | 71 | 31 | 2 | 5.57 | 37 |
| 8 | 1.78 | 2.07 | 66 | 32 | 2 | 5.65 | 36 |
| 9 | 1.78 | 2.15 | 65 | 33 | 2 | 5.73 | 35 |
| 10 | 1.78 | 2.23 | 64 | 34 | 2 | 5.81 | 34 |
| 11 | 1.78 | 2.31 | 62 | 35 | 2 | 5.89 | 33 |
| 12 | 1.78 | 2.39 | 60 | 36 | 2.03 | 5.92 | 32 |
| 13 | 1.78 | 2.47 | 58 | 37 | 2.06 | 5.95 | 31 |
| 14 | 1.78 | 2.55 | 57 | 38 | 2.09 | 6.06 | 30 |
| 15 | 1.78 | 4.71 | 55 | 39 | 2.16 | 5.97 | 29 |
| 16 | 1.78 | 4.63 | 55 | 40 | 2.22 | 6.03 | 27 |
| 17 | 1.78 | 4.47 | 53 | 41 | 3.04 | 5.01 | 14 |
| 18 | 1.78 | 4.39 | 52 | 42 | 3.04 | 5.25 | 7 |
| 19 | 1.81 | 4.42 | 51 | 43 | 3 | 5.37 | 6 |
| 20 | 1.84 | 4.45 | 50 | 44 | 3 | 5.45 | 4 |
| 21 | 1.84 | 4.37 | 49 | 45 | 3 | 5.53 | 3 |
| 22 | 1.87 | 4.4 | 48 | 46 | 3 | 5.61 | 2 |
| 23 | 1.9 | 4.43 | 48 | 47 | 3 | 5.69 | 0 |
| 24 | 1.9 | 4.27 | 46 |  |  |  |  |

Table S2. Major parameters of the selected meta-atoms arranging on the metalens

In fact, Our design method is not limited by the polarization state of incident light. Specifically, we designed a LWIR polarization-insensitive achromatic metalens for six selected wavelengths within the bandwidth, i.e. 8, 9, 10, 10.6, 11, 12 μm. and the relative group delay in each zone is bounded by 60 fs. The diameter of the metalens is 3.18 cm and *F* number is 1. Figure S2a gives the 2D optical intensity profiles of the designed unpolarized metalenses along the z-axis, the actual focal length almost keeps at the preset value of 3000λ_0_ as marked by the yellow-dashed lines, and the normalized focal length shift is less than 2.5×10^-4^. The intensity distribution curves along the z-axis crossing the center of each focal spot are presented in Figure S2b, and the peak intensity are observed near the focal plane. The depth of focus for the selected wavelengths are varies from 6.95λ_0_ to 9.33λ_0_. Figure S2c gives the intensity distribution curves at the preset focal plane, and the values of *FWHM* varies from 1.0λ_0_ to 1.27λ_0_, which shows diffraction limited focusing performance. To meet the requirement of phase and dispersion engineering, a library of Si meta-atoms was created by varying their geometric parameters, i.e., length *L* of square, radius *R* of circle, and *R_in_* and *R_out_* of ring, as shown in Figure S2d. Considering the maximum high-aspect ratio limited by the achievable fabrication technology, the optimized height *H* and pitch *P* are 7 and 7.25 μm, respectively. The phase and group delay distribution of the library is depicted in Figure S2e. Considering appropriate matching errors of phase and group delay and selecting meta-atoms arranged on the metalens’ surface, a 3.18-cm-diameter unpolarized achromatic metalens can be achieved with aspect ratio of meta-atoms less than 7. Figure S2f gives the optical intensity profiles along the propagation direction for the 6-sampled wavelengths, which gives clear evidence for actual achromatic focusing.


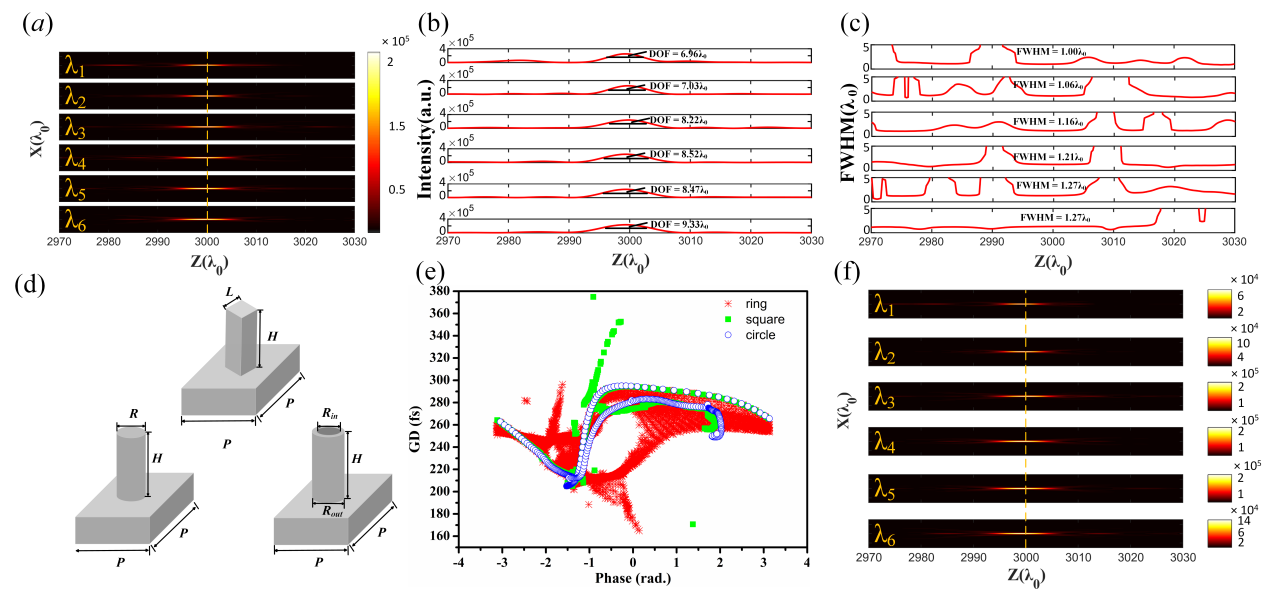


Figure S2. (a) 2D optical intensity profiles of the designed unpolarized achromatic metalenses with relative group delay of 60 fs for 6-sampled wavelengths. (b) Corresponding intensity distribution curves and (c) focal spot sizes in the z-axis crossing the center of each focal spot. The values of DOF and *FWHM* at the preset focal plane for different wavelengths are also marked beside the curves. (d) Polarization insensitive meta-atoms, i.e., square, circle and ring silicon pillars standing on a silicon substrate. (e) Phase and group delay distribution of the library. (f) Optical intensity profiles of the unpolarized achromatic metalens with aspect ratio of Si meta-atoms less than 7.

For the 3.18-cm-diameter unpolarized achromatic metalens, the entire surface is divided into 2194 concentric rings, 198 zones are created according to phase- dispersion engineering, and 1050 kinds of meta-atoms are selected for arranging within theses zones. Table S3 lists the major parameters of partial meta-atoms, including the inner radius and outer radius. Figure S3 gives the phase and amplitude transmittance distributions. These meta-atoms can achieve the required phase coverage of -π~π, while the amplitude transmittance varies from 0.3~0.9.

Table S3. Major parameters of partial ring silicon pillars arranging on the metalens

| No. | *R_in_*(μm) | *R_out_*(μm) | GD(fs) | ... | No. | *R_in_*(μm) | *R_out_*(μm) | GD(fs) |
| --- | --- | --- | --- | --- | --- | --- | --- | --- |
| 1 | 1.11 | 0.61 | 205 |  | 1027 | 1.56 | 0.76 | 265 |
| 2 | 1.11 | 0.61 | 205 |  | 1028 | 1.56 | 0.76 | 265 |
| 3 | 1.11 | 0.61 | 205 |  | 1029 | 1.56 | 0.76 | 265 |
| 4 | 1.11 | 0.61 | 205 |  | 1030 | 1.56 | 0.76 | 265 |
| 5 | 1.11 | 0.61 | 205 |  | 1031 | 1.56 | 0.76 | 265 |
| 6 | 1.17 | 0.69 | 206 |  | 1032 | 1.56 | 0.76 | 265 |
| 7 | 1.17 | 0.69 | 206 |  | 1033 | 1.56 | 0.76 | 265 |
| 8 | 1.17 | 0.69 | 206 |  | 1034 | 1.98 | 1.38 | 265 |
| 9 | 1.36 | 1.24 | 206 |  | 1035 | 1.98 | 1.38 | 265 |
| 10 | 1.36 | 1.24 | 206 |  | 1036 | 1.98 | 1.38 | 265 |
| 11 | 1.23 | 0.77 | 206 |  | 1037 | 1.98 | 1.38 | 265 |
| 12 | 1.09 | 0.97 | 206 |  | 1038 | 1.98 | 1.38 | 265 |
| 13 | 1.05 | 0.93 | 206 |  | 1039 | 1.98 | 1.38 | 265 |
| 14 | 1.46 | 1.02 | 207 |  | 1040 | 1.98 | 1.38 | 265 |
| 15 | 1.19 | 1.07 | 207 |  | 1041 | 1.98 | 1.38 | 265 |
| 16 | 1.44 | 1 | 207 |  | 1042 | 1.98 | 1.38 | 265 |
| 17 | 1.44 | 1 | 207 |  | 1043 | 1.98 | 1.38 | 265 |
| 18 | 1.44 | 1 | 207 |  | 1044 | 1.98 | 1.38 | 265 |
| 19 | 1.44 | 1 | 207 |  | 1045 | 1.98 | 1.38 | 265 |
| 20 | 1.44 | 1 | 207 |  | 1046 | 1.98 | 1.38 | 265 |
| 21 | 1.44 | 1 | 207 |  | 1047 | 1.98 | 1.38 | 265 |
| 22 | 1.44 | 1 | 207 |  | 1048 | 1.98 | 1.38 | 265 |
| 23 | 1.44 | 1 | 207 |  | 1049 | 1.98 | 1.38 | 265 |
| 24 | 1.44 | 1 | 207 |  | 1050 | 1.98 | 1.38 | 265 |

**
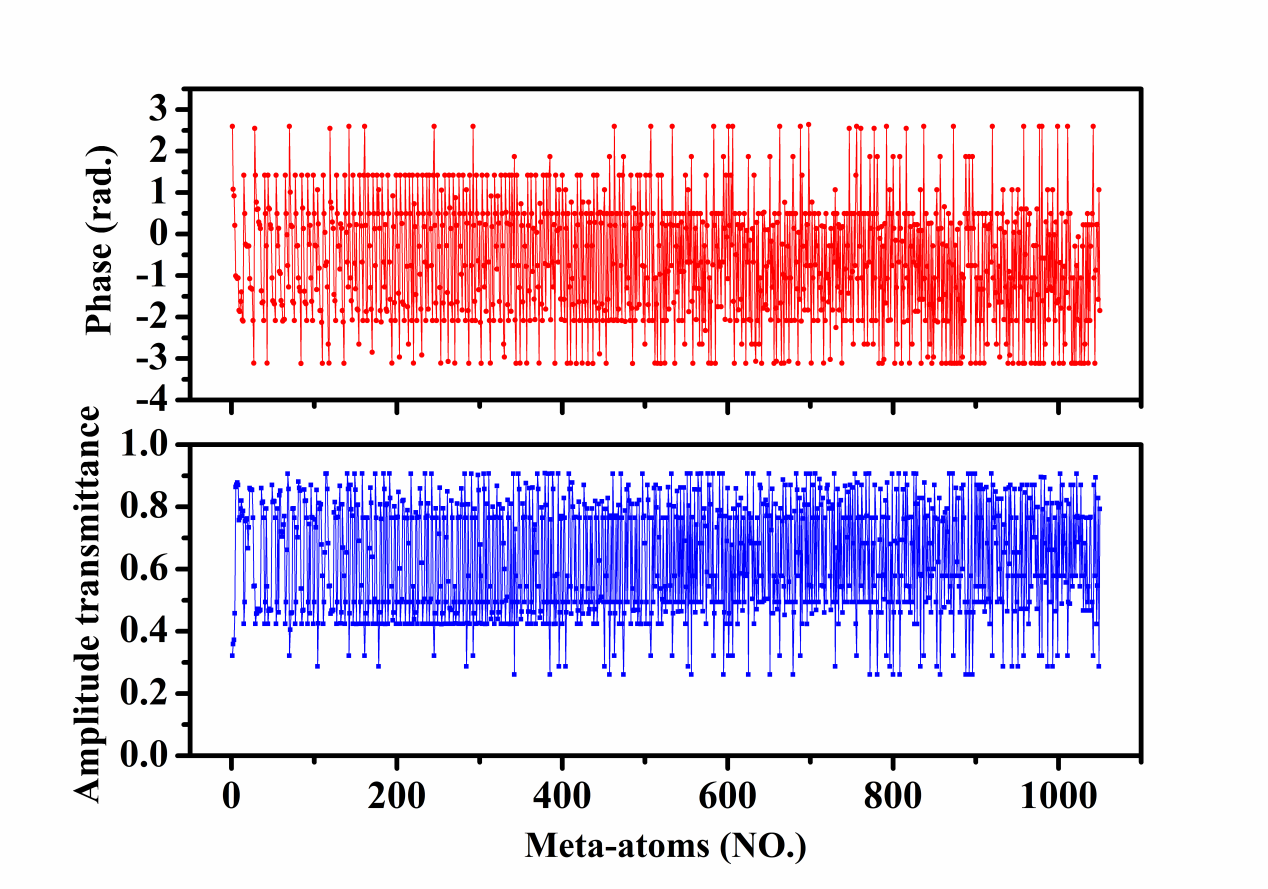
**

Figure S3. Phase and amplitude transmittance distributions of the 1050 ring silicon pillars.

**Supplementary Note S3: Sample fabrication and measurement setup**

The large-aperture LWIR achromatic metalenses are fabricated on a 400 μm thick double-polished silicon wafer. A 200 nm thick layer of silicon dioxide is deposited on the silicon wafer using plasma enhanced chemical vapor deposition (PECVD). The silicon oxide surface is uniformly coated photoresist and patterned with meta-atoms. A SiO_2_ mask is constructed by reactive ion etching machine (RIE), the photoresist residue is stripped, and the mask is then transferred to the bulk silicon to a depth of 6.8 μm by inductively coupled plasma (ICP) etching with a substrate temperature of -110 ℃. The SiO_2_ residue is stripped with buffered oxide etch solution, and the fabricated metalenses are ready for characterization. This processing flow is shown in Figure S4. In fact, with the aid of the SiO_2_ mask, the surface of the finished microstructure is smooth. In addition, the weight of fabricated metalens is about 5.4 g.


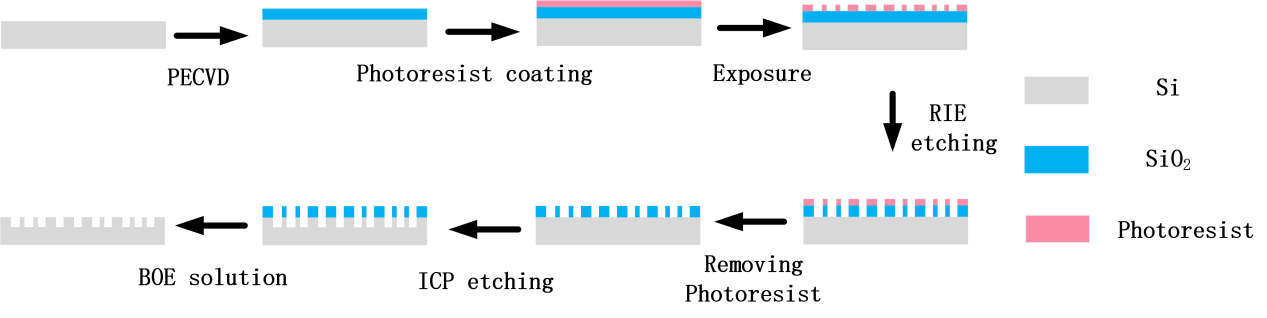


Figure S4. Microfabrication flow for fabricating the proposed achromatic metalenses.


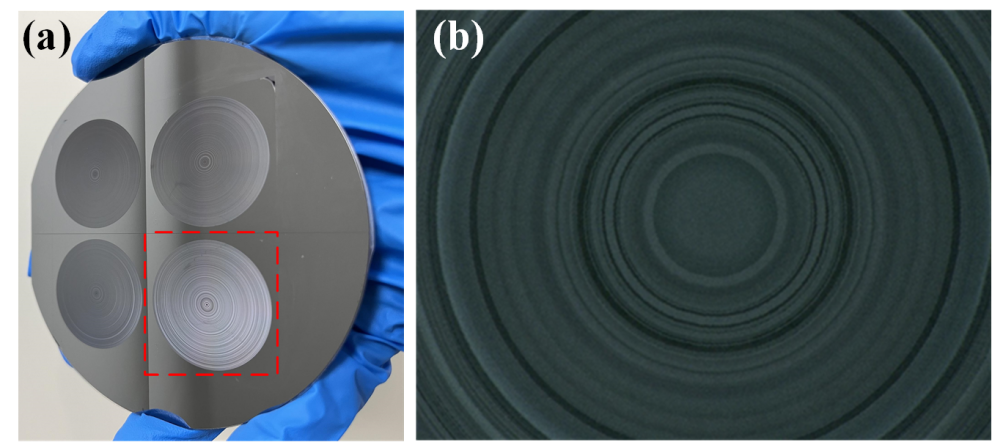


Figure S5. (a) Optical images of the achromatic metalenses with diameter of 3.18 cm. (b) optical microscope zoom-in images of the lens marked by the red dashed box in a.


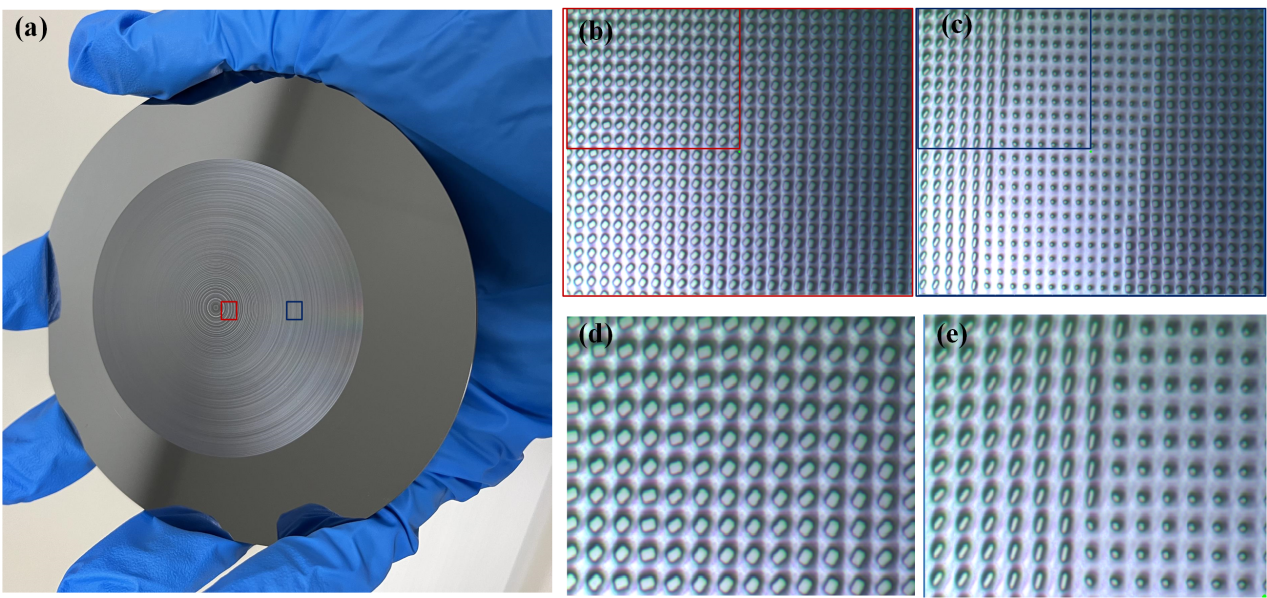


Figure S6. (a) Optical image of the 6.36-cm-diameter achromatic metalens (b-c) Zoom-in images of the metalens bounded by the red b) and blue c) rectangles, respectively. (d-e) Further enlarged images corresponding to the top left corner of the images in b-c.

The schematic of the measurement setup is illustrated in Figure S7. The metalens was illuminated with 10.6 μm light emitted from a CO_2_ laser. After passing through a reflectively adjustable attenuator, the optical beam was incident on a plano-convex lens with focal length of 12.7 mm (#39-542, Edmund), where the reflect light was absorbed by the heat absorber. The diameter of the beam was enhanced to approximately 40 mm by a 90° off-axis parabolic mirror with an effective focal length of 177.8 mm (#35-537, Edmund). Then the reflect beam was sent through a linear polarizer, impinged on the substrate of the achromatic metalens, and the optical field was obtained by a long wave infrared CCD (M3J1280) with a spatial resolution of 1280×1024 and pixel size of 12×12 μm^2^, which was mounted on a motorized translation stage (EM-LSS65-30C1) with a step of 2 μm.


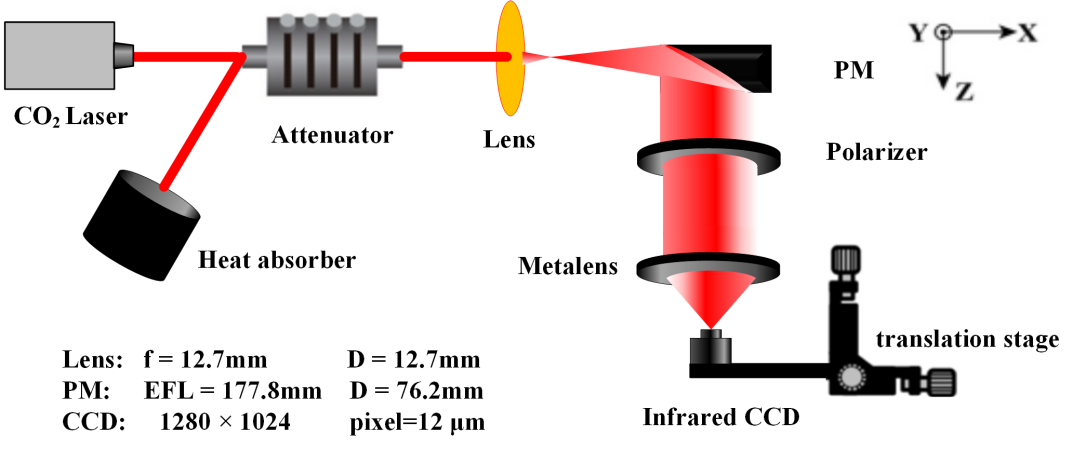


Figure S7. Schematic illustration of the measurement setup.

**References:**

[1] W. T. Chen, A. Y. Zhu, V. Sanjeev, M. Khorasaninejad, Z. Shi, E. Lee, F. Capasso, *Nat. Nanotechnol.* **2018**, *13*, 220.

[2] Z. Li, P. Lin, Y. W. Huang, J. S. Park, W. T. Chen, Z. Shi, C. W. Qiu, J. X. Cheng, F. Capasso, *Sci. Adv.* **2021**, *7*.

[3] Z. Li, R. Pestourie, J. Park, Y. Huang, S. G. Johnson, F. Capasso, *Nat. Commun.* **2022**, *13*, 2409.

[4] N. Li, Z. Xu, Y. Dong, T. Hu, Q. Zhong, Y. H. Fu, S. Zhu, N. Singh, *Nanophotonics* **2020**, *9*, 3071.

[5] A. M. Baracu, C. A. Dirdal, A. M. Avram, A. Dinescu, R. Muller, G. U. Jensen, P. C. V. Thrane, H. Angelskår, *Micromachines* **2021**, *12*, 501.

[6] M. Y. Shalaginov, S. An, Y. Zhang, F. Yang, P. Su, V. Liberman, J. B. Chou, C. M. Roberts, M. Kang, C. Rios, Q. Du, C. Fowler, A. Agarwal, K. A. Richardson, C. Rivero-Baleine, H. Zhang, J. Hu, T. Gu, *Nat. Commun.* **2021**, *12*, 1225.

[7] M. Hou, Y. Chen, J. Li, F. Yi, *Sci. Adv.* **2024**, *10*, o4847.

[8] A. Li, H. Duan, H. Jia, L. Long, J. Li, Y. Hu, *J. Optics* **2024**, *26*, 65005.
